# A soft photopolymer cuboid that computes with binary strings of white light

**DOI:** 10.1038/s41467-019-10166-4

**Published:** 2019-05-24

**Authors:** Alexander D. Hudson, Matthew R. Ponte, Fariha Mahmood, Thomas Pena Ventura, Kalaichelvi Saravanamuttu

**Affiliations:** Department of Chemistry and Chemical Biology, McMaster University, 1280 Main St. West, Hamilton, ON L8S 4M1 Canada

**Keywords:** Computational science, Computer science, Polymers

## Abstract

Next-generation stimuli–responsive materials must be configured with local computational ability so that instead of a discrete on-off responsiveness, they sense, process and interact reciprocally with environmental stimuli. Because of their varied architectures and tunable responsiveness to a range of physical and chemical stimuli, polymers hold particular promise in the generation of such “materials that compute”. Here, we present a photopolymer cuboid that autonomously performs pattern recognition and transfer, volumetric encoding and binary arithmetic with incandescent beams. The material’s nonlinear response to incident beams generates one, two or three mutually orthogonal ensembles of white-light filaments, which respectively self-organize into disordered, 1-D and 2-D periodic geometries. Data input as binary (dark-bright) strings generate a unique distribution of filament geometries, which corresponds to the result of a specific operation. The working principles of this material that computes with light is transferrable to other nonlinear systems and incoherent sources including light emitting diodes.

## Abstract (web summary)

Some next-generation computing may be based in physical systems that respond directly and reciprocally to environmental stimuli. Here, the authors describe a photoresponsive material that autonomously performs computations with incident beams of incoherent white light.

## Introduction

Soft polymer thin films, monolayers, colloids, fluids, gels and solids are essential for the design of stimuli–responsive systems and devices^1–3^. When stimulated by electromagnetic, electrical, chemical or mechanical signals, these pliant polymer architectures transition between states while exhibiting discrete changes in physical or chemical properties that can be harnessed for biosensing^4^, controlled drug delivery^5^, tuning photonic band gaps^6,7^, surface wettability and swelling^8^. The ultimate objective of this field is the biomimicry of intelligent responsiveness such as tactility, vision, camouflage, contractility and flight where complex natural sensors such as skin, eye and muscle seamlessly adapt to environmental stimuli through exquisitely programmed response sequences^9^. Artificial intelligent sensing systems could be developed by configuring local computational ability into soft polymer constructs and in this way, enabling autonomous sensing, manipulation and processing of stimuli signals without the need for external processors or electrical power. A recent and seminal example of such a polymeric “material that computes” coupled the nonlinear self-oscillations generated through the Belousov–Zhabotinsky reaction in a group of gels with piezoelectric (PZ) cantilevers to perform binary pattern recognition^10^.

**Fig. 1:**
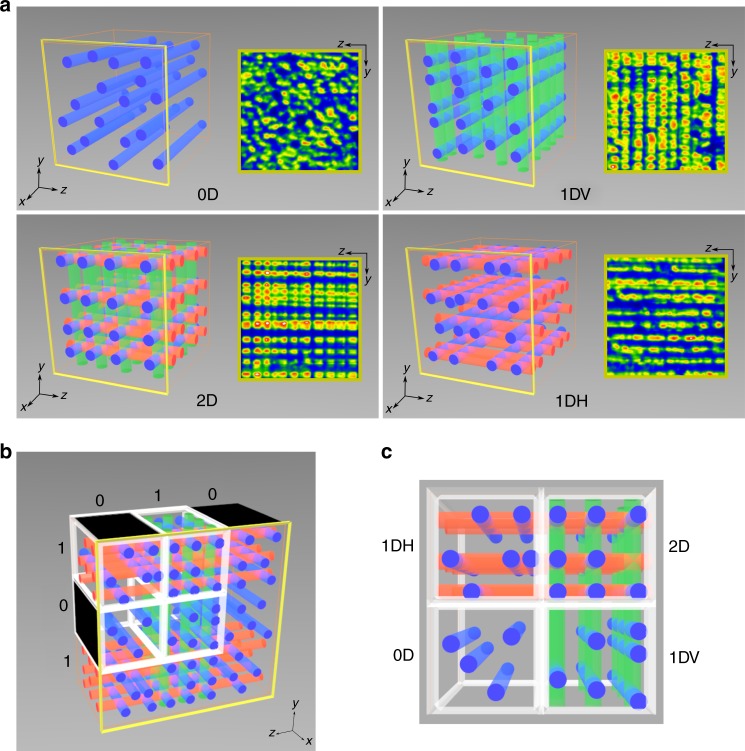
Working principles of the photopolymer cuboid. Schemes and actual spatial intensity profiles of **a** disordered (0D), one-dimensionally ordered (1DH, 1DV), and two-dimensionally ordered (2D) configurations of carrier filaments at the output face (*yz* plane) of the polymer cuboid. **b** Simultaneous input of binary strings along the *y* and *z* axes divides the cuboid into voxels, each containing a specific configuration of carrier filaments. **c** The output of voxels containing the four possible filament configurations

Here, we present a self-contained, soft photoresponsive system—comprising only a homogeneous photopolymerizable fluid contained in a transparent cuboid and up to three beams of white incandescent light—that performs multiple operations including the recognition and transfer of binary data, single-step volumetric encoding, and two foundational operations of digital computing—the addition and subtraction of binary strings (Fig. 1, Supplementary Movie 1). These operations are performed by large ensembles of nonlinear optical waves, specifically soliton-like filaments, which originate from the nonlinear response of a photopolymerizing medium to incident optical fields^11–13^. Binary data input into the cuboid as black and bright stripes of light precisely control the 3-D spatial distribution and self-organization of filaments and in this way, generate the result of an operation, which can be read in a single step at the cuboid output face. Because it is based on nonlinear waves, which are unified through mathematical expression and exhibit strikingly similar dynamics in disparate media^14^, this system provides proof of a concept that is transferrable to a wide range of nonlinear materials with photoresponses that can be tuned in terms of reversibility, speed, and magnitude^15,16^. Equally powerfully, the system functions with incoherent white light—like sunlight—comprising all visible wavelengths and is therefore also operable with compact, inexpensive and visible light-emitting diodes (LEDs), which typically emit incoherent light.

## Results

### Working principles

Figure 1 and Supplementary Movie 1 depict the working principles of our system, which consists of dense populations (~ 8000 cm^−2^)^11^ of white-light filaments confined to a polymer cuboid (~ 1 cm^3^). Up to three mutually orthogonal populations of filaments are generated through the modulation instability (MI)—stochastically driven filamentation^11,12,15,17,18^—of broad incandescent beams (Δ*λ* = 370 nm–1000 nm) launched along the *x*, *y*, and *z* axes of the cuboid, which contains a photopolymerizable organosiloxane fluid^11,13,19,20^. Mechanisms underlying the MI of optical fields in this and other polymerizable systems have been detailed elsewhere^11,17,18,21,22^ and are briefly described below.

When launched into the cuboid, a broad incandescent beam with a uniform spatial intensity profile initiates free-radical polymerization along its propagation axis. Corresponding refractive index changes (Δ*n*) push the nascent system into the nonlinear regime where noise—normally insignificant and random intensity variations—becomes amplified. As these intensity modulations grow, the beam becomes unstable and collapses—typically over 20 min—into large populations of statistically equivalent filaments (see Methods). Each filament induces a high-refractive index channel along its path, populates this cylindrical waveguide as bound optical modes and travels through the medium without diverging. Because the photopolymerization process is irreversible, the waveguides are permanently inscribed in the medium.

Two properties of MI-induced filaments are critical to our system: akin to spatial solitons, the filaments are discrete, non-divergent beams that form only under nonlinear conditions. Under linear conditions, by contrast, they would blur and rapidly become indistinguishable from each other due to the significant natural divergence of incandescent light. Secondly, because they originate from spatially and temporally incoherent light^11–13,15,17–19,21,22^, the filaments do not suffer optical interference despite their close proximity. Specifically, the spatial coherence length of the optical fields is ~ 0.31 μm, which is significantly smaller than the typically 75 μm width of the self-trapped filament^11^. It is also important to note that MI of an incandescent beam can be elicited in the photopolymerizable fluid because of its relatively slow response time, which spans seconds to minutes. The system therefore responds to the time-averaged intensity profile of the incandescent fields, which characteristically possess temporal coherence lengths at the femtosecond scale^11^. Such dense populations of non-interfering, discrete filaments would be extremely challenging to achieve with coherent laser light.

The operations performed by this system are based on four distinct types of spatial ordering, which originate from the collective interactions and spontaneous self-organization of filament populations^13^ (Fig. 1a): because they are seeded by noise, filaments generated through MI of a single, broad beam are randomly distributed in space and appear at the cuboid exit face as a disordered population of high-intensity spots, which we represent as 0D. Two mutually orthogonal filament populations—generated through simultaneous MI of two beams—spontaneously organize into a periodic stack of grids, which appear as rows of spots at the output. Depending on the propagation axes of the beams, rows are periodically stacked along the horizontal or vertical directions and denoted 1DH and 1DV, respectively. Three mutually orthogonal populations, generated through simultaneous MI of three beams, self-organize into a cubic lattice; the output, in this case, is a square array of spots (2D). The mechanisms underlying the self-organization of filaments were previously elucidated in detail^13^ and are briefly summarized here: self-organization originates from the strong propensity of neighboring filaments to maximize the number of orthogonal collisions; this is because the resulting intersection points between filaments—where there is optimal overlap of their respective optical fields—generate the maximum changes in refractive index and consequently, attract, and confine the greatest amount of optical intensity. In the case of two orthogonal populations, the maximum number of collisions is achieved when constituent filaments intersect their orthogonal counterparts to form grids, which can be stacked vertically or horizontally and yield the 1DH and 1DV configurations, respectively. The same mechanism of pairwise orthogonal intersections drives the dynamics of three mutually orthogonal filament populations, which achieve the maximum number of orthogonal collisions by self-organizing into a cubic lattice giving rise to the 2D configuration^13^. Self-organization occurs only when participating beams possess comparable intensities and simultaneously suffer MI. Under these conditions, orthogonal beams experience equivalent nonlinear changes in refractive index and consequently, possess similar dynamics; at early, nascent times, this enables filaments to mutually shift in space to maximize collisions.

For the operations, we designate the filament population propagating along *x* as carriers, which are operated on by their orthogonal counterparts—encoders—traveling along *y* and *z*. Data are input by patterning the beams launched along *y* and *z* with a binary sequence (string) of bright and dark stripes (bits). Because MI-induced generation of encoders occurs exclusively in the bright bits, the input of binary strings effectively divides the polymer cuboid into micro-volumes—voxels—in which carriers encounter 0, 1, or 2 encoder populations and therefore, respectively, adopt the 0D, 1DH, 1DV or 2D configuration (Fig. 1b). The consequent distribution of carrier configurations in the voxels provides the result of an operation, which is output at the exit face (*yz* plane) of the cuboid as a unique combination of the quaternary configurations.

### Recognition and transfer of a binary string of light

**Fig. 2:**
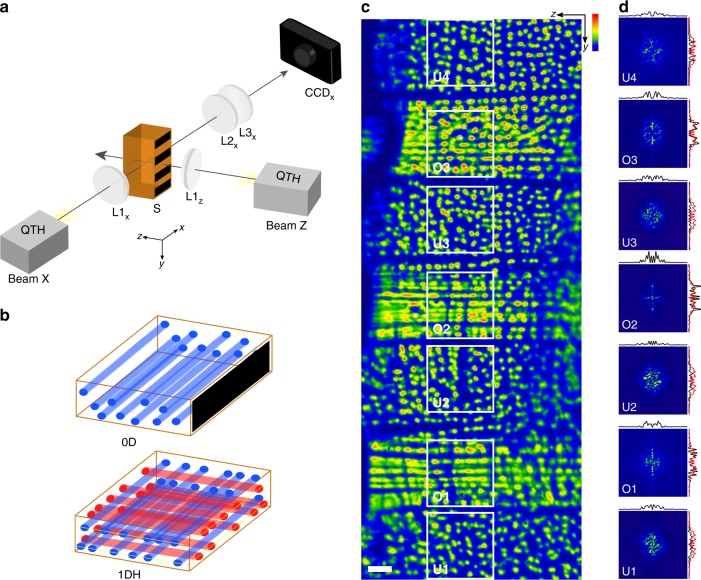
Recognition and transfer of a binary string of light. A binary string comprising **a** seven dark and bright stripes (bits) is launched through Beam Z into the polymer cuboid (S). Beam Z is emitted by a quartz–tungsten halogen (QTH) lamp and collimated through planoconvex lens L1_*z*_. The spatial intensity profile of Beam X (emitted by a QTH lamp and collimated through planoconvex lens L1_*x*_) at the output (*yz*) plane is imaged through a pair of planoconvex lenses (L2_*x*_, L3_*x*_) onto a charge coupled device (CCD) camera. The output string consists of seven stripes of **b** alternating 0D and 1DH configurations, which **c** corresponds to the input binary string (scale bar = 200 μm; blue (minimum) to red (maximum) intensity scale provided). **d** Fast Fourier Transform (FFT) spectra of areas of the output contained in white squares. 1D vertical and horizontal profiles acquired through the origin are provided in black; for reference, averaged 1D vertical profiles of the entire spectrum are provided in red

The first operation that we demonstrate is the recognition of a string of binary data by the system and its transfer to an orthogonal beam (Fig. 2). Here, Beam X (Fig. 2a) has a uniform intensity profile while Beam Z is patterned with the binary string of seven stripes (*N* *=* 7) with alternating dark (intensity < threshold for MI) and bright (intensity > threshold for MI) intensity (Fig. 2b). When launched into the photopolymerizable medium, the entire Beam X suffers MI and generates carriers propagating along *x* throughout the cuboid. However, MI of Beam Z and generation of encoders along *z* are confined to rectangular prismic volumes—voxels—defined by its bright stripes. Carrier filaments traveling along *x* therefore encounter (and self-organize with) encoders traveling along *z* only within these voxels; they remain disordered everywhere else (Fig. 2b). The output of the carriers (Fig. 2c) is a binary sequence of seven stripes with alternating configurations of disordered (0D) and one-dimensionally ordered (1DH) spots. The output is read in a single step and quantified through Fast Fourier Transform (FFT) analysis, which reveals high frequency peaks only for the ordered 1DH configuration (Fig. 2d, Supplementary Note 1). The output exactly matches the binary string input through Beam Z where the dark and bright stripes correspond to the 0D and 1DH states, respectively and confirms its transfer to the initially uniform Beam X.

### Single-step volumetric encoding of optical data

**Fig. 3:**
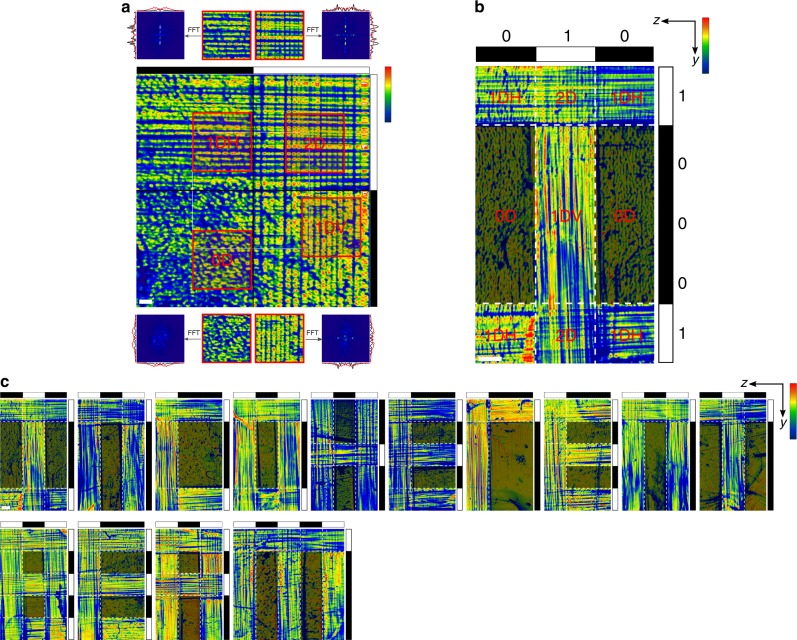
Single-step volumetric encoding in the photopolymer cuboid. Output face (*yz* plane) of polymer cuboid when **a** encoded by two identical binary strings 0 (black bar) 1 (white bar) introduced along the *y* and *z* axes. The four carrier configurations at the output were quantified through Fast Fourier Transform (FFT) analysis of select regions indicated by red squares; **b** when encoded by binary strings 010 and 10001 to generate the letter “I”. Carrier configurations are labeled; **c** when encoded with different binary string pairs to generate words “incoherent beam”. (**a**–**c** blue (minimum) to red (maximum) intensity scales are provided; scale bar = 500 μm; **b**–**c** the 0D configuration has been dulled to guide the eye.)

The system also performs active operations—where input data are processed to yield a result—by employing both orthogonal populations of encoders (Fig. 3 and Supplementary Fig. 1). An active operation generates a unique 3-D distribution of carrier configurations within the cuboid; this result is output at the exit face as a specific combination of the quaternary (0D, 1DH, 1DV, and 2D) carrier configurations. The first active operation that we demonstrate is the single-step volumetric encoding of optical data in the polymer cuboid. Here, two independent binary strings input respectively through Beams Y and Z combine within the polymer cuboid and divide it into voxels. All voxels are populated by carriers but contain different numbers (0, 1, or 2) of encoder populations, and therefore, different carrier configurations. This is different from conventional 3-D optical data storage, which must employ high-intensity, two-photon processes to access volumetric control, and writing of binary patterns in the photochromic material is carried out element by element^23^. Equally important, the read-out in our system is also a single-step, non-destructive process and is based on the output of waveguides. By contrast, conventional techniques typically rely on the luminescence of chromophores for the element-by-element read-out process, which moreover can lead to photobleaching of encoded information.

In the simplest case of encoding (Fig. 3a), Beams Y and Z are each patterned with a binary string of one bright and one dark stripe (*N* = 2), whereas Beam X remains uniform. MI occurs everywhere in Beam X to form carriers but encoders form only within the bright stripes of Beams Y and Z and divide the cuboid into quadrants or four cubic voxels. In the upper left voxel, carriers encounter encoders only along *z* and assume configuration 1DH, whereas in the lower right voxel, they adopt the 1DV configuration owing to equivalent interactions with encoders along *y*. Carriers remain disordered in configuration 0D in the lower left voxel where neither Beam Z nor Beam Y suffers MI. In the upper right voxel, all three filament populations self-organize into a cubic lattice and thereby the 2D configuration. At the *yz* output plane, the distinct configurations of the carrier filaments are quantitatively confirmed through FFT spectra (Fig. 3a).

To demonstrate the versatility of the encoding operation, we generate and then read out a sequence of alphabet letters that spell “incoherent beam” (Fig. 3b, c). The letters are volumetrically encoded in the cuboid and generated through a specific pair of orthogonal binary strings input through Beams Z and Y. For example, the two binary strings—010 and 10001—operate on carrier filaments to generate the letter “I” within the cuboid (Fig. 3b). The two horizontal strokes of the letter are defined by the 1DH configuration, which results from the combination of the “1” bit in Beam Z (which generates encoders along z) and “0” bit in Beam Y (which suppresses MI along *y*). Conversely, the vertical stroke of “I” corresponds to the 1DV configuration of carriers, resulting from the combination of the “1” bit in Beam Y (which generates encoders along *y*) and “0” bit in Beam Z (which suppresses MI along *z*). At the intersections of the horizontal and vertical strokes, carriers adopt the 2D configuration due to the combination of “1” bits from both Beams Y and Z (generating encoders along both *y* and *z*). The encoded letter can be visualized in real space and through FFT, distinguished from the background 0D configuration (the latter has been dulled in Fig. 3b, c as a guide to the eye).

### Binary arithmetic

**Fig. 4:**
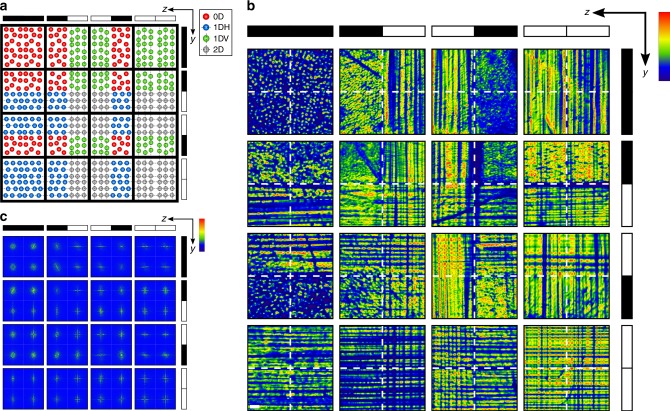
Output generated by pairs of two-bit strings. **a** Expected and corresponding **b** experimental results of the 16 (2^2^ × 2^2^) patterns generated at the polymer cuboid output (*yz* plane) by all combinations of a pair of 2-bit binary strings introduced along the *y* and *z* axis. Each output pattern comprises different combinations of carrier configurations 0D (red), 1DH (blue), 1DV (green), and 2D (gray), which can be quantified through **c** corresponding FFT spectra of experimental results. (Bits 0 and 1 are represented by white and black bars, respectively; scale bar in **b** = 200 μm; in **b**, **c**, blue (minimum) to red (maximum) intensity scales are provided.)

The quaternary nature of its output increases the capacity of our system by 2^*N*^-fold compared with the conventional binary string of electrical bits. Given integral number *N* = number of bits, an electrical binary string with *N* = 2 allows 2^2^ = 4 possible combinations, i.e., 00, 10, 01, and 11. By contrast and as depicted in Fig. 4a, two such binary strings can be simultaneously input into our system to generate 2^2^ × 2^2^ = 2^4^ = 16 combinations of the quaternary configurations 0D, 1DH, 1DV, 2D. These predicted combinations exactly match experimental results (Fig. 4b and Supplementary Fig. 2), which are quantitatively confirmed in corresponding FFT spectra (Fig. 4c, Supplementary Figs. 2 and 3). The output combination in our system can be read in a single step and quantified (and therefore digitized) through FFT analysis (Supplementary Movie 2).

**Fig. 5:**
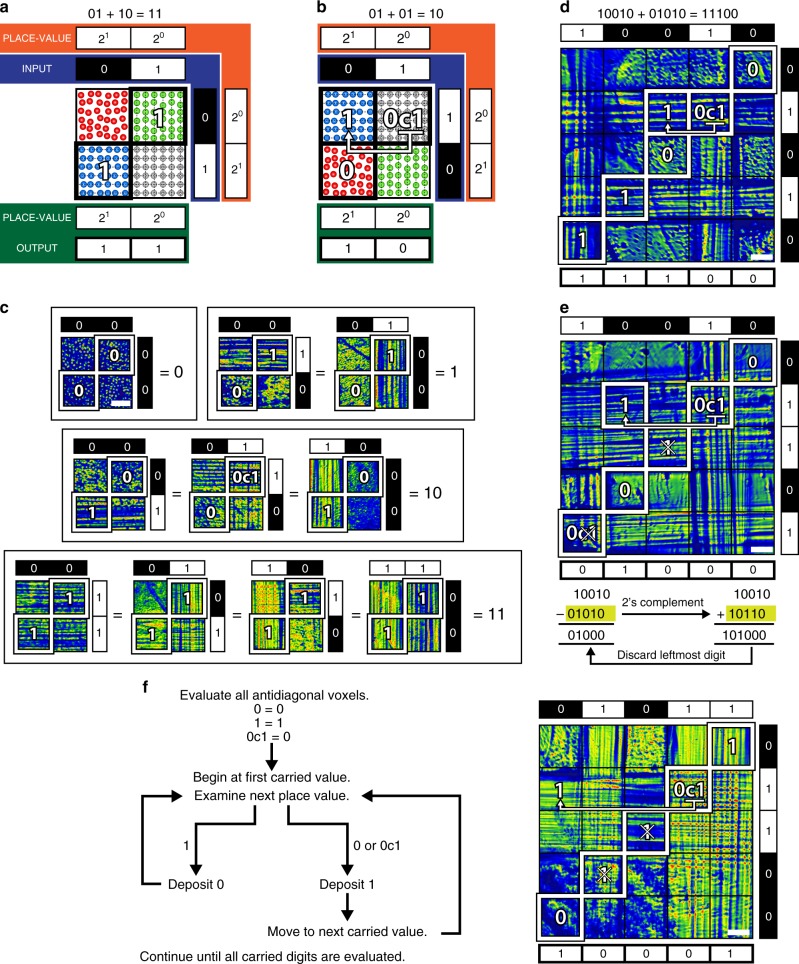
Binary arithmetic. Schemes of addition of **a** 01 + 10 and **b** 01 + 01. Addends are input as binary strings through Beams Y and Z; place values are indicated. The result is contained in the antidiagonal of the output (*yz* plane). Carrier configurations 0D (red spots) = 0, 1DV (green spots), 1DH (blue spots) = 1, 2D (gray spots) = 0C1. **c** Addition is commutative and different addend pairs generate different output patterns but the same sum; examples from the 2^2^ × 2^2^ system are tabulated. Any addend may be added on the grid, provided that the sum does not exceed *N* digits for a 2 ^*N*^ × 2 ^*N*^ grid. Shorter binary strings are accommodated by appending leading zeroes as shown in **d** for the sum of 10010 and 1010 performed on a 2^5^ × 2^5^ system. Subtraction is accomplished by adding the two’s complement of the minuend. We determine **e** the difference between 10010 and 1010 by adding 10110 and dropping the leftmost digit. This occurs naturally as the bottom–left voxel signifies a carried digit that cannot be contained within the grid. **f** A flowchart summarizing the steps of an addition algorithm including the treatment of carried over digits as exemplified in the accompanying output pattern corresponding to the sum of 01011 and 00110. (**c**–**f** scale bar = 500 μm)

Most significantly, its quaternary output enables the system to perform two fundamental operations of binary arithmetic—addition and subtraction (Fig. 5). The scheme in Fig. 5a depicts the addition of two binary strings, 10 and 01, input, respectively, through Beams Y and Z; in all cases, MI of uniform Beam X generates carriers throughout the cuboid. The place value of the binary strings increases from right to left for Beam Y and from top to bottom for Beam Z. The strings divide the cuboid into voxels, each of which contains 0, 1, or 2 encoder populations that operate on carriers and generate a unique output. The sum in binary form is read from the antidiagonal of the output; each element is the result of the operations of encoders with the same place value on the carriers. To interpret results, we assign a value of 0 to configuration 0D, 1 to 1DH and 1DV, and 0 carry 1 (0C1) to 2D. In Fig. 5a, the addition of 01 and 10 generates an antidiagonal of two elements comprising the 1DV and 1DH configurations, respectively. This correctly represents a sum of 11. For the addition of 0 1 and 0 1 shown in Fig. 5b, the first element of the antidiagonal reads “0C1” and the carried value is read from the off-diagonal as 1. Because the second element of the antidiagonal is 0, the sum is 10. Experimental examples of addition in the 2^2^  × 2^2^ system (Fig. 4) are tabulated in Fig. 5c and classified according to the absolute value of the sum. Because the addition of different pairs of numbers can have the same sum, the 10 tabulated patterns correspond to four unique results. Interestingly, although addition is commutative, our system has the ability to distinguish between the 1DH and 1DV configurations, which both correspond to “1”. This allows the identity of the input binary strings to be elucidated from the result.

The system is scalable. For a 2^*N*^ × 2 ^*N*^ system, the maximum computable sum = 2 ^*N*^ − 1. For example, in the 2^5^ × 2^5^ system shown in Fig. 5d, the largest possible sum is 31. We use this system to demonstrate the addition of 10010 and 01010 to yield a sum of 11100. Using the same system, we also demonstrate the subtraction of the same numbers (Fig. 5e). In the case of the latter, we employ the radix complement method employed in digital computing (Supplementary Note 2)^24^. The addition of 00110 + 01011 to yield 10001 in Fig. 5f illustrates the treatment of carried over digits, which is summarized in the accompanying flowchart. Specifically, a carried over digit can only be deposited at the nearest element of increased place value that possesses a value of 0 or 0C1. When carried over an element possessing a value of 1, a result of 0 is deposited at that place value (further details and additional examples are provided in Supplementary Fig. 6).

The system employs a two-step process to perform addition: the two addend binary strings are input simultaneously and the output containing the result including carried digits is read in a single step. This contrasts with digital computing where addition is performed by adders consisting of sequences of transistor-based logic gates^24^. An individual full-adder determines the sum of three binary inputs, which typically comprise two addends and a carried value^24^. *N* full-adders are required to find the sum of two *N*-digit numbers. In a typical carry lookahead device, pairs of digits with the same place value are first analyzed to calculate carried values. The sums at each place value including any carried values are then determined in parallel^24^. Both our system and digital computing employ the two’s complement method to perform subtraction, enabling the same hardware to be employed for addition and subtraction operations^24,25^.

## Discussion

We have shown that a self-contained, single-component, light-responsive system performs sophisticated operations on incident optical fields without relying on external electrical power or processors. The system responds to low intensity, incandescent light, which—similar to ambient sunlight—comprises all visible wavelengths and is moreover, spatially and temporally incoherent. Interestingly, this system is reminiscent of the light-guiding-light model proposed by Snyder^26^ more than two decades ago in which the pairwise interactions of spatial solitons propagating through a photoresponsive monolith forms the basis of logic operations. Because of the characteristic universality in dynamics and interactions of nonlinear waves, the working principles of this system is applicable to other nonlinear materials. Moreover, because it operates with incandescent light, the system should be operable at all the visible wavelengths. For example, we have already shown  that the 16 distinct output patterns of the 2^2^ × 2^2^ system can be generated with miniature blue LEDs (Supplementary Fig. 4).

We note that the transferability of its working principles to other nonlinear materials is a critical and powerful feature of our system. For the current proof-of-concept study, we employed a photopolymerizable medium that undergoes permanent changes in refractive index at a relatively slow rate. In addition, an experiment carried out with a pair of 10-bit binary strings suggests that the spatial resolution of this particular system reaches its limit at ~100 voxels (Supplementary Fig. 5); although the voxels are clearly delineated, there is mild deviation of filaments at the voxel boundaries. This deviation could increase if strings > 10 bits are employed and impede the accurate determination of results. Ongoing work includes developing soft materials with rapidly reversible photoresponses and the ability to elicit self-trapped filaments with smaller widths. The corresponding increase in the filament population would increase the capacity—i.e., the number of voxels—available for operations. By working with reversibly responsive systems, multiple operations could be carried out in a single-photoresponsive cuboid.

## Methods

### Preparation of photopolymerizable sols

Photopolymerizable organosiloxane sols were prepared through a previously-described method^11,13^. Briefly, the acid catalyzed hydrolysis and condensation of 3-methacryloxypropyltrimethoxysilane (17.6 g) was initiated through the addition of 0.05 m HCl (1.1 g). The homogenized sol was then sensitized to visible light with the photoinitiator, bis(η^5^cyclopentandienyl) bis(2,6-difluoro-3-(1H-pyrrol-yl)-phenyl) titanium(IV) (0.096 g, *λ*_max_ = 460 nm) and stirred for at least 24 h. The resulting sol, which was transparent and orange in color, was protected from ambient light and filtered through a polytetrafluoroethlyene membrane (pore size = 0.2 μm) immediately prior to use.

### Optical assembly

All optical experiments were carried out on the custom-built assembly depicted in Supplementary Fig. 1. Three incandescent (quartz–tungsten halogen) lamps (*λ* = 370 nm−1000 nm) were positioned along the *x*, *y*, and *z* axes and each collimated with a planoconvex lens (f.l. = 250 mm) prior to being launched into the photopolymer cuboid sample. Typical optical powers for the beam propagating along *x* was 0.5 mW and 2.5–3.0 mW for those propagating along *y* and *z*. The spatial intensity profile of the beam propagating along *x* was imaged through a planoconvex lens pair (f.l. = 250 mm) onto a high-resolution charge coupled device (CCD) camera. The photopolymer cuboid consisted of the organosiloxane sol contained in a transparent, glass cuvette (10 mm × 10 mm × 5 mm); the cuvette was sealed with a glass coverslip to ensure that all entrance faces were optically flat. For computing operations, vinyl amplitude masks representing binary strings were attached to the entrance faces of the cell. A typical experiment spanned ∼ 20 min, which corresponded to the time required for filaments to achieve a steady-state, stationary configuration^13^.

## Supplementary information

Supplementary Information

Description of Additional Supplementary Files

Supplementary Movie 1

Supplementary Movie 2

## Data Availability

The data that support the findings of this study are available from the corresponding author upon reasonable request.
