# Supplementary material for: A soft photopolymer cuboid that computes with binary strings of white light
Source: Nat Commun. 2019 May 24;10:2310. doi: 10.1038/s41467-019-10166-4 (PMC6534534; doi:10.1038/s41467-019-10166-4)
Supplement: Supplementary file 1 — Supplementary Information [file 41467_2019_10166_MOESM1_ESM.pdf]

Supplementary Information

A soft photopolymer cuboid that computes with binary strings of white light

Alexander D. Hudson[†], Matthew R. Ponte[†], Fariha Mahmood, Thomas Pena Ventura and Kalaichelvi Saravanamuttu*

Department of Chemistry and Chemical Biology, McMaster University, 1280 Main St. West, Hamilton, Ontario, Canada

[†]These authors contributed equally to the work

*Correspondence to:
Dr. Kalaichelvi Saravanamuttu
Associate Professor
Department of Chemistry and Chemical Biology
McMaster University
1280 Main St.West, Hamilton
Ontario L8S 4M1,
CANADA
Email: kalai@mcmaster.ca

Supplementary Note 1: Acquisition and analysis of Fast Fourier Transform (FFT) spectra

Each operation generates a distinct spatial intensity profile at the output (*yz* face) of the photopolymer cuboid, which in turn corresponds to a specific distribution of voxels (Supplementary Figure 1). For example, in the case of pattern recognition and transfer of a single binary string (Figure 2), the cuboid output comprises a series of horizontal stripes, each containing a distinct filament configuration (i.e., 0D or 1DH). The input of two binary strings as in the case of encoding (Figure 3) or binary arithmetic (Figures 4-5) operations results in more complex patterns comprising square and rectangular regions, each containing one of four distinct filament configuration (i.e., 0D, 1DH, 1DV or 2D). Two dimensional FFT of selected square regions within individual voxels (e.g. Figure 4) quantitatively confirmed the filament configurations.

To demonstrate that the readout process could be digitized, we performed further FFT analysis on the cuboid output. For a given output of the photopolymer cuboid, we first selected square regions within each voxel. Figure 2b, 3a and Supplementary Figure 2 display regions that were selected in experiments of pattern recognition transfer and optical encoding, respectively. These sample regions were converted into square matrices (e.g. 60 x 60 or 100 x 100) of optical intensity. After the minimum intensity was subtracted from all elements to remove background noise, the Fourier function in the Wolfram Mathematica software suite was applied to each element. Absolute values of the real portion of the transform were then collected. To resolve high frequency peaks, the minimum value in this new matrix was subtracted from each element and its dominant peak at frequency = 0, set to zero.

For a given output of a particular system (e.g. 2^2 x 2^2), we analyzed the central one-dimensional horizontal and vertical cross-sections of FFT spectra in each voxel. Each output – consisting of four voxels - therefore generated four horizontal and four vertical FFT traces resulting in 64 different traces for the entire system. To determine order along a particular axis, each FFT trace was compared to a threshold spectrum. The latter was generated by averaging all one-dimensional FFT spectra (along either the horizontal or vertical direction) acquired from voxels that did not contain the 0D configuration. (We deliberately excluded voxels that did not contain at least 1D ordering of filaments in order to set a significant threshold in determining order). The presence of order along a particular axis was then determined by the appearance of discrete peaks with amplitudes that significantly exceeded the threshold value. Representative examples of spectra employed for this analysis are collected in Supplementary Figure 3.

Supplementary Note 2: Radix complement method

The Radix complement method was adapted from the two's complement method, which is employed to represent signed integers in digital computing (*1*, *2*). The radix complement of any N-digit number z in base-b is $b^N - z$. In binary or base-2, this is the two's complement. To determine the difference between two positive integers y and z, $(y - z)$, we first obtain the two's complement of z, denoted z'. The binary strings y and z' are input into the material and the resulting pattern is interpreted as a sum. This sum, $(y + z')$, is the two's complement of the difference. It can be readily converted to the desired difference by discarding the leftmost (most significant) digit. If this operation is performed on the smallest possible grid (i.e. an N by N grid where N = the number of digits of the largest input value), this leftmost bit naturally overflows the boundaries of the grid and is therefore automatically discarded. For cases where z and y have

a different number of digits, leading zeroes are added to the smaller number, and both are treated as N-digit values. This method is applicable for differences $(y - z) \geq 0$, where $y, z \geq 0$.

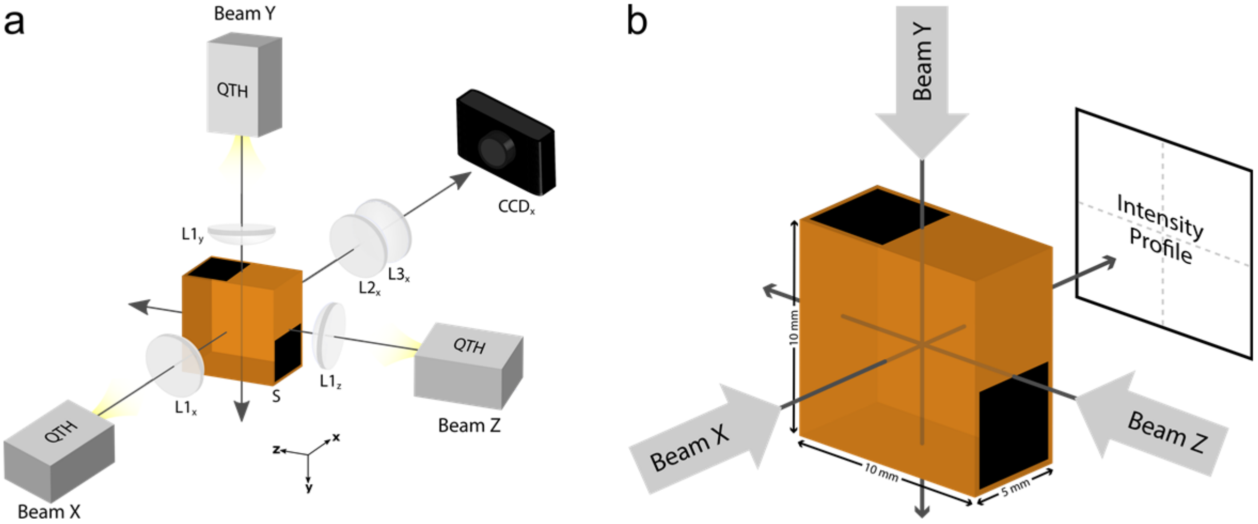

Supplementary Figure 1 Scheme of optical assembly employed for computing operations of the photopolymer cuboid. (a) Three incandescent light beams emitted by quartz-tungsten halogen (QTH) lamps positioned along the *x*, *y* and *z* axes were each collimated by a planoconvex lens (L_1) and launched into the glass cuvette containing photopolymerizable sol (S). The spatial intensity profiles of Beam X and Beam Z at the respective exit faces of this photopolymer cuboid was imaged through a pair of planoconvex lenses (L_2, L_3) onto a charge-coupled device (CCD) camera. (a) A magnified scheme shows that vinyl amplitude masks attached to entrance faces of S modulate Beams Y and Z to represent 01 and 10, respectively.

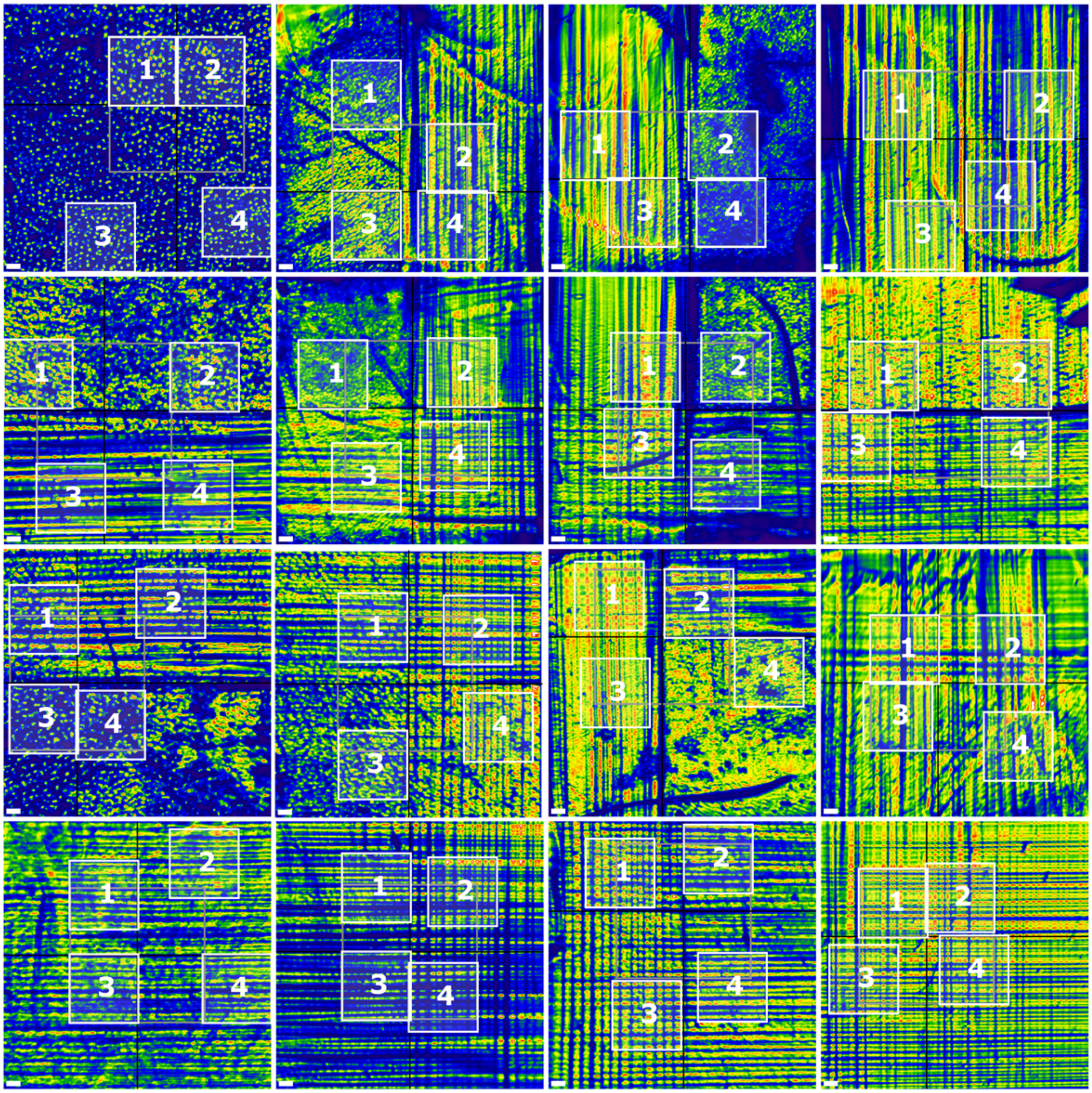

Supplementary Figure 2 Output generated by two-bit string pairs. The 16 patterns generated at the output of the polymer cuboid in the 2^2 x 2^2 system each consisted of four quadrants. Each quadrant contained a distinct filament configuration, which was determined through Fast Fourier Transforms (FFT). The four sample regions employed for FFT analysis in each pattern are indicated within white squares. (Scale bar = 200 μm). See also Figure 4.

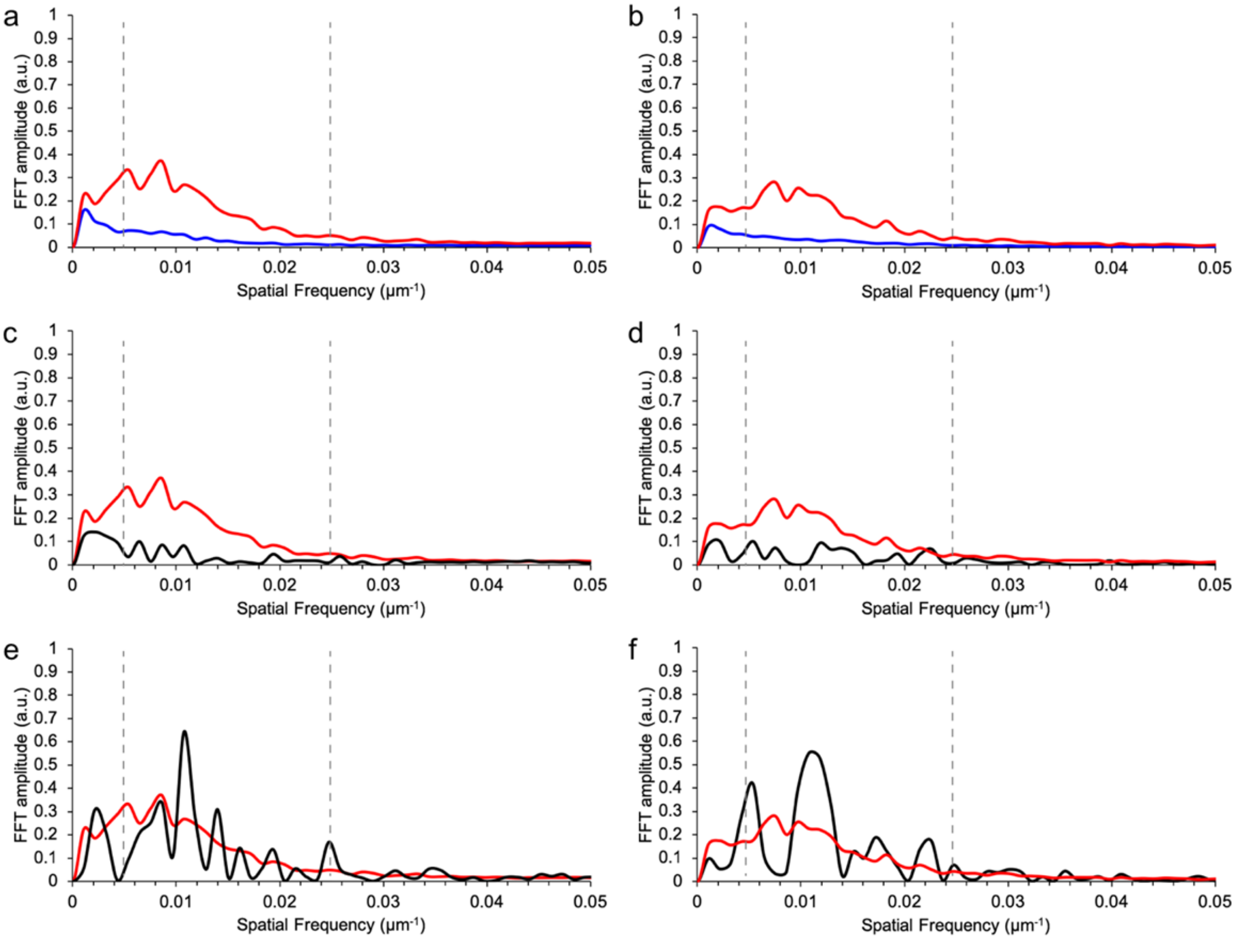

Supplementary Figure 3 Fast Fourier Transform (FFT) analysis of the output generated by strings 01 (Beam Z) and 01 (Beam Y) (Figure 3a). One-dimensional FFT traces along the (a, c, e) horizontal and (b, d, f) vertical directions. The averaged FFT trace of all regions with order along the (a, c, e) horizontal or (b, d, f) vertical directions is provided in red. The averaged FFT trace for regions without order along the (a) horizontal and (b) vertical directions is provided in blue. A single FFT trace (black) of a region with no order along the (c) horizontal and (d) vertical directions shows that it falls beneath the threshold of the respective averaged FFT trace (red). A single FFT trace (black) of a region with order along the (e) horizontal and (f) vertical directions show several discrete peaks that significantly exceed the amplitude of the respective averaged FFT trace (red). (Gray dotted lines indicate the reciprocal of filament diameters).

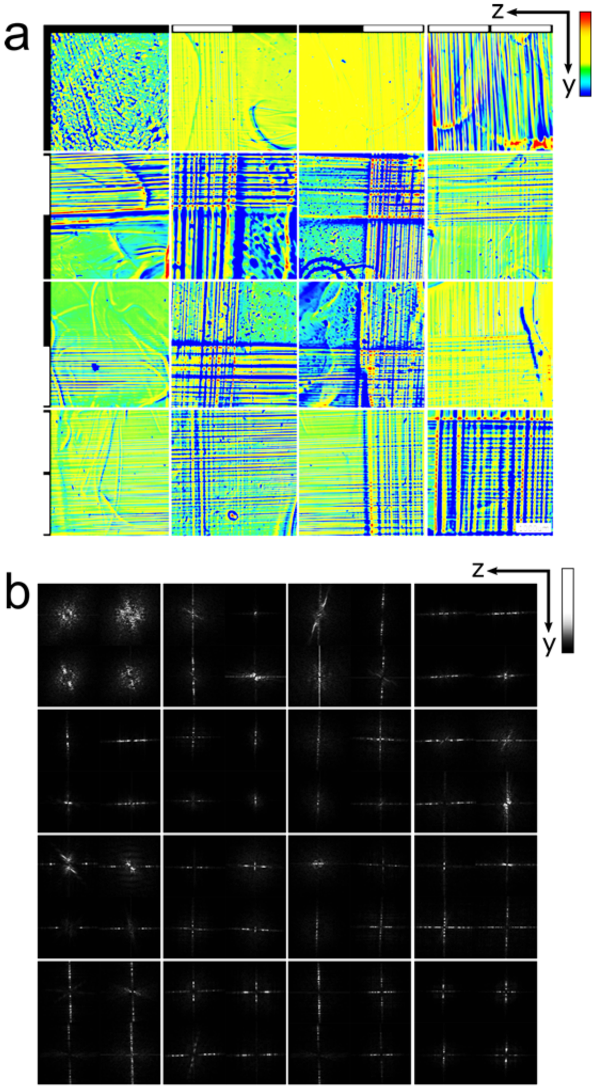

Supplementary Figure 4 Computing with LED beams. (a) Experimental results of the 16 (2^2x2^2) patterns generated at the polymer cuboid output (*yz* plane) by all combinations of a pair of 2-bit binary strings introduced along the *y* and *z* axis of LED beams. (b) Corresponding Fast Fourier Transform spectra. The contrast of images has been enhanced for clarity.

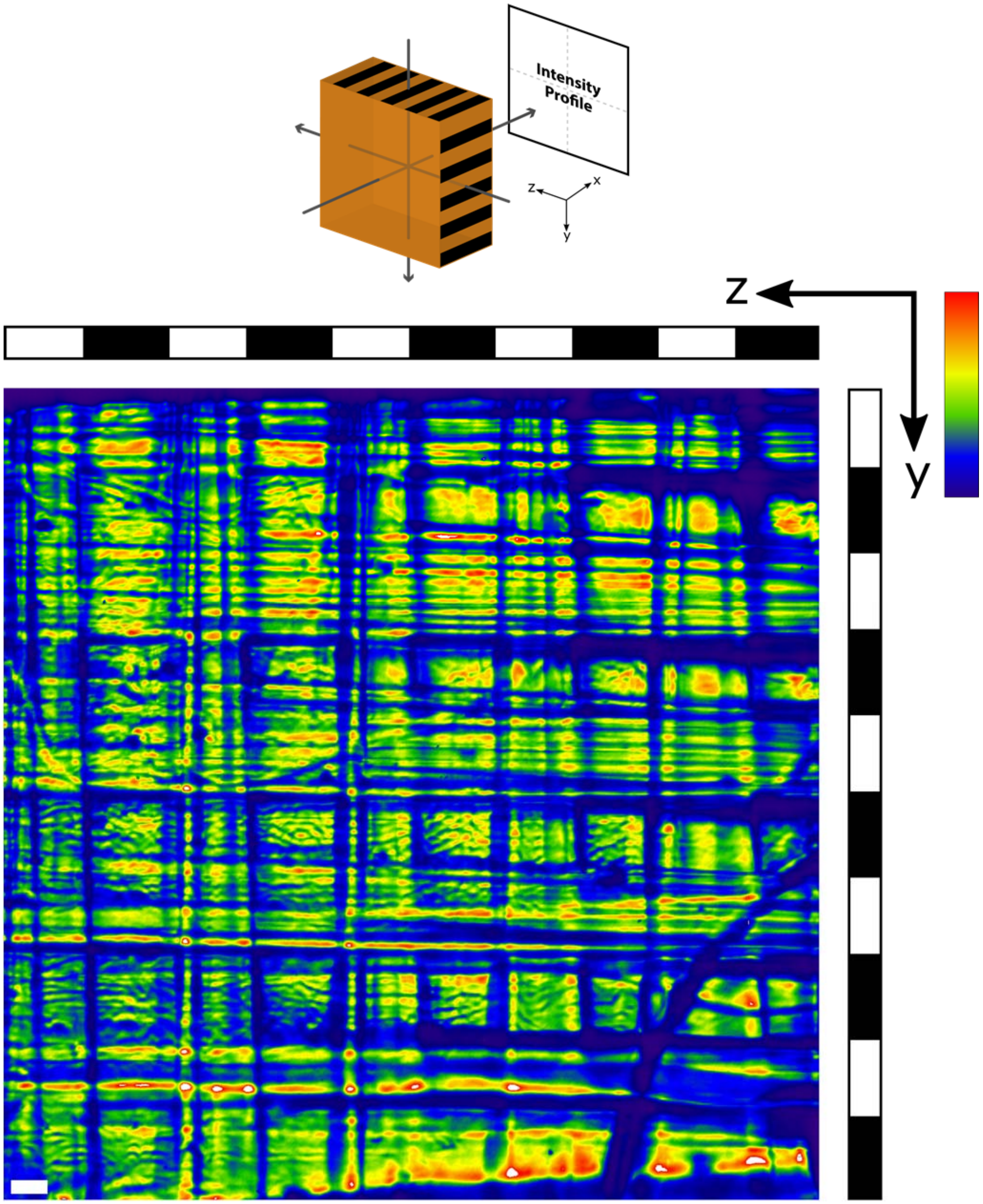

Supplementary Figure 5 Spatial intensity profile at the cuboid output generated from the interaction of binary strings, 1010101010 and 0101010101. A scheme of the experiment and laboratory coordinates is included.

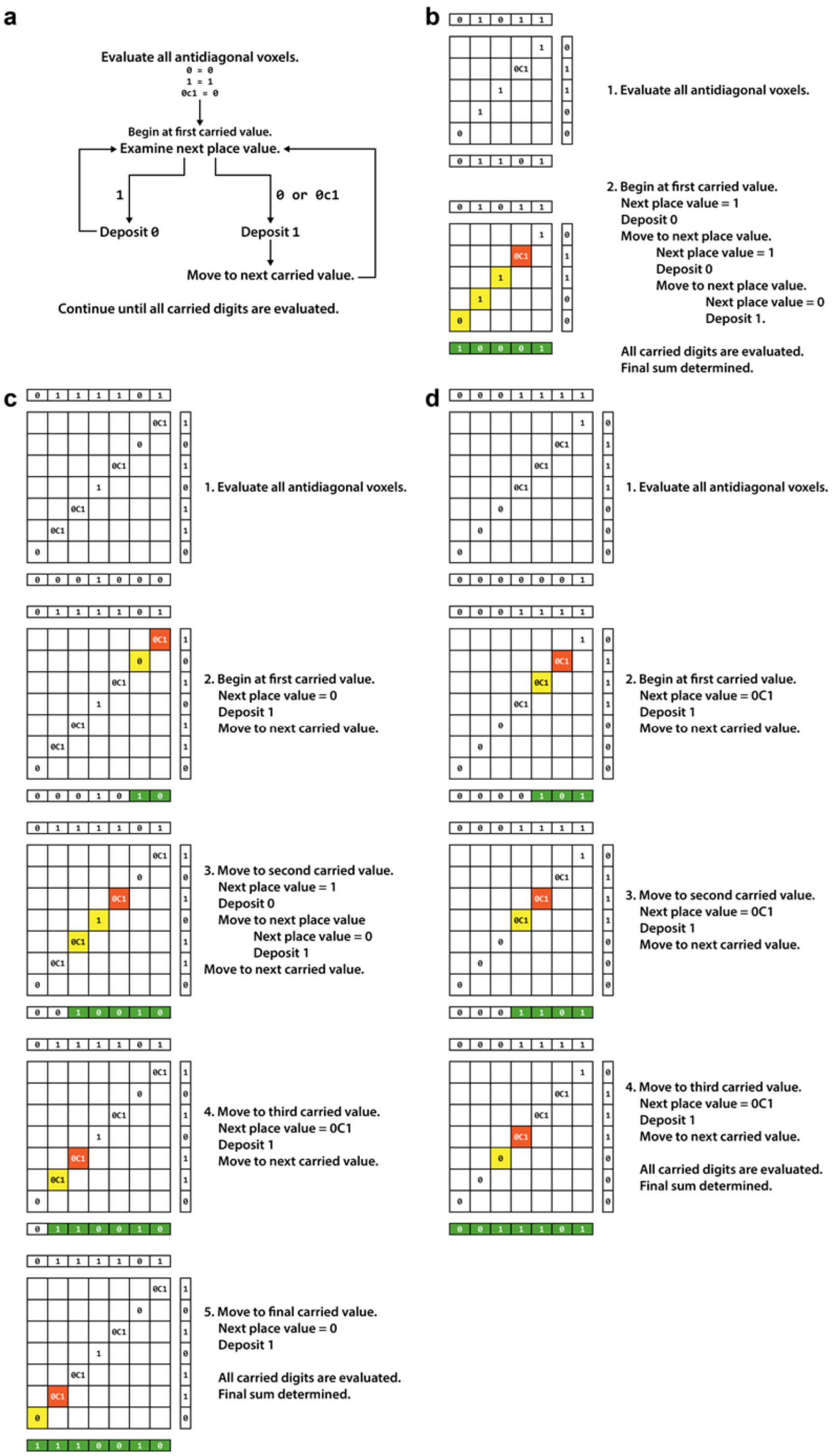

Supplementary Figure 6 (a) Flow-chart summarizing the process of evaluating a binary sum from the output obtained in Figure 5f. The sum (b) 01011 + 00110 is used to demonstrate how carried digits are propagated over multiple positions. The sum (c) 0111101 + 0110101 shows cases of carried digits that precede values of 0, 1 and 0C1. Finally, the sum (d) 000111 + 0001110 shows how multiple, sequential carried digits are evaluated.

Supplementary References

1. J. Seiffertt, *Digital Logic for Computing* (Springer International Publishing, Cham, Switzerland, 2017).

2. J. G. Brookshear, *Computer Science: An Overview* (Pearson Education, Inc., Boston, Massachusetts, ed. 8, 2003).
