# Supplementary material for: A soft photopolymer cuboid that computes with binary strings of white light
Source: Nat Commun. 2019 May 24;10:2310. doi: 10.1038/s41467-019-10166-4 (PMC6534534; doi:10.1038/s41467-019-10166-4)
Supplement: Supplementary file 2 — Description of Additional Supplementary Files [file 41467_2019_10166_MOESM2_ESM.pdf]

Description of Additional Supplementary Files

File Name: Supplementary Movie 1
Description: Working principles of the photopolymer cuboid that computes.

File Name: Supplementary Movie 2
Description: Example of FFT-based analysis, which would enable digitization of cuboid output. From left to right are the cuboid output, corresponding FFT spectra and result of FFT threshold analysis. The quaternary filament configurations are assigned integer values: 0D = 0, 1DH = 1, 1DV = 2 and 2D = 3. Three different output patterns of the 23 x 23 were analyzed, with their expected patterns stored as "S", "C", and "I", respectively. If the read-out matches a pattern in a database, the matching response is output; if there is no match "?" is displayed.
